# A Context-Specific Role for Retinoblastoma Protein-Dependent Negative Growth Control in Suppressing Mammary Tumorigenesis

**DOI:** 10.1371/journal.pone.0016434

**Published:** 2011-02-22

**Authors:** Sarah M. Francis, Subrata Chakrabarti, Frederick A. Dick

**Affiliations:** 1 London Regional Cancer Program, London, Ontario, Canada; 2 Department of Biochemistry, University of Western Ontario, London, Ontario, Canada; 3 Department of Pathology, University of Western Ontario, London, Ontario, Canada; 4 Children's Health Research Institute, London, Ontario, Canada; University of Medicine and Dentistry of New Jersey, United States of America

## Abstract

**Background:**

The ability to respond to anti-growth signals is critical to maintain tissue homeostasis and loss of this negative growth control safeguard is considered a hallmark of cancer. Negative growth regulation generally occurs during the G0/G1 phase of the cell cycle, yet the redundancy and complexity among components of this regulatory network has made it difficult to discern how negative growth cues protect cells from aberrant proliferation.

**Methodology/Principal Findings:**

The retinoblastoma protein (pRB) acts as the final barrier to prevent cells from entering into the cell cycle. By introducing subtle changes in the endogenous mouse *Rb1* gene (*Rb1^ΔL^*), we have previously shown that interactions at the LXCXE binding cleft are necessary for the proper response to anti-growth signals such as DNA damage and TGF-β, with minimal effects on overall development. This disrupts the balance of pro- and anti-growth signals in mammary epithelium of *Rb1^ΔL/ΔL^* mice. Here we show that *Rb1^ΔL/ΔL^* mice are more prone to mammary tumors in the *Wap-p53^R172H^* transgenic background indicating that negative growth regulation is important for tumor suppression in these mice. In contrast, the same defect in anti-growth control has no impact on *Neu*-induced mammary tumorigenesis.

**Conclusions/Significance:**

Our work demonstrates that negative growth control by pRB acts as a crucial barrier against oncogenic transformation. Strikingly, our data also reveals that this tumor suppressive effect is context-dependent.

## Introduction

Maintenance of tissue homeostasis is a tightly regulated process and the loss of responsiveness to negative growth signals can alter this delicate balance, leading to cancer [1]. This is especially evident in the breast, where epithelial cells undergo cycles of quiescence, proliferation, differentiation, and apoptosis during the menstrual cycle and as a result of pregnancy [2]. Tight negative growth control of the mammary epithelial compartment is crucial, and disruption of the balance between mitogenic and anti-growth signals can leave this tissue susceptible to the formation of cancer [3], [4]. Therefore, delineating how negative growth control of breast epithelial cells is lost during tumor formation is essential to understand the pathogenesis of breast cancer.

Insensitivity to negative growth signals is considered a hallmark of cancer cells [1]. This means that the mechanism that conveys growth inhibiting signals from DNA damage or transforming growth factor β (TGF-β) to the cell cycle machinery is universally disrupted. In general, growth inhibiting signals arrest the cell cycle by blocking cyclin dependent kinase (CDK) activity through the actions of cyclin dependent kinase inhibitors (CKIs) such as p15Ink4b, p16Ink4a, p21Cip1, and p27Kip1 [3], [5]. This in turn activates the retinoblastoma protein (pRB). In cell culture, disruption of an individual CKI, such as p21Cip1, can result in deregulated proliferation despite signals from negative growth regulatory stimuli such as DNA damage [6], [7]. However, negative growth regulators such as TGF-β are capable of inducing a cell cycle arrest in the absence of any one of these CKI proteins [8], [9], [10], [11]. Furthermore, ablation of individual CKI genes in mice has no effect on viability and is accompanied by surprisingly few developmental abnormalities. Interestingly, each knockout mouse strain develops tumors in a specific subset of tissues, but none of these mice develop mammary tumors [11], [12], [13], [14], [15], [16], [17], [18]. In fact, pRB itself was previously considered dispensable for mammary development [19]. This is surprising because pRB pathway components such as cyclin D1 are commonly amplified, and pRB itself is sometimes lost in human breast cancers and these are direct targets of CKI regulation [20], [21], [22], [23], [24]. Thus, even though the current body of literature suggests that anti-growth effects greatly influence mammary epithelial cells [1], mouse models reveal layers of complexity that make it challenging to understand how negative growth responses protect mammary epithelial cells from aberrant proliferation and ultimately tumorigenesis.

Activation of pRB maintains cells in G0/G1 and prevents inappropriate cell cycle entry [3]. Breast epithelial cells frequently respond to anti-growth signals from DNA damage, exogenous growth factors like TGF-β, and other cellular stresses. All of these serve to activate pRB and arrest proliferation [25]. Using a knock-in mouse model with a discrete defect in the pocket domain of *Rb1* (*Rb1^ΔL^*), we have reported the remarkable discovery that pRB uses interactions with LXCXE-motif-containing proteins almost exclusively for growth arrest in response to stressful negative growth stimuli, and this mechanism is largely dispensable for cell cycle control in development allowing us to obtain viable animals with this defect [26], [27]. The lone circumstance in development where pRB interactions with LXCXE containing proteins, such as histone deacetylases, are critical is in mammary epithelium. In this tissue, ductal morphology and branching are normal, but there is mild hyperplasia of the luminal cell compartment [26]. Our data indicates that, the *Rb1^ΔL^* mutation results in an inability to silence transcription of E2F target genes and renders cells insensitive to anti-growth signals, including TGF-β, DNA damage, and other senescence cues [26], [27]. Furthermore, cells from these mice fail to induce growth arrest in response to ectopic expression of the CKI proteins p16Ink4a and p21Cip1 [26]. This suggests that there are likely many stress-induced negative growth signals to which cells from these mice are uniquely resistant. Since pRB-dependent negative growth control is dramatically reduced in the mammary glands of *Rb1^ΔL^* mice, but viability and overall development are normal, these animals provide a unique opportunity to examine the impact of negative growth signals in suppressing mammary cancer.

In order to understand the role of pRB LXCXE-dependent negative growth control during mammary tumorigenesis, we have crossed our mice with a *Wap-p53^R172H^* transgenic strain. *Wap- p53^R172H^*; *Rb1^ΔL/ΔL^* females developed mammary tumors more frequently and at a significantly younger age than control mice, while showing no differences in histopathological characteristics or metastasis. This strongly suggests that loss of negative growth regulation in this model exacerbates cancer development at a very early stage. In contrast, co-expression of *Neu* and *Rb1^ΔL^* did not accelerate mammary tumor initiation, progression, or metastatic dissemination. Surprisingly, the contrasting data between the two transgenic mammary tumor models indicates that failure to respond to negative growth signals by pRB affects cancer incidence in a context-dependent manner.

## Results

### pRB-LXCXE interactions act as an initial barrier to tumor formation

**Figure 1:**
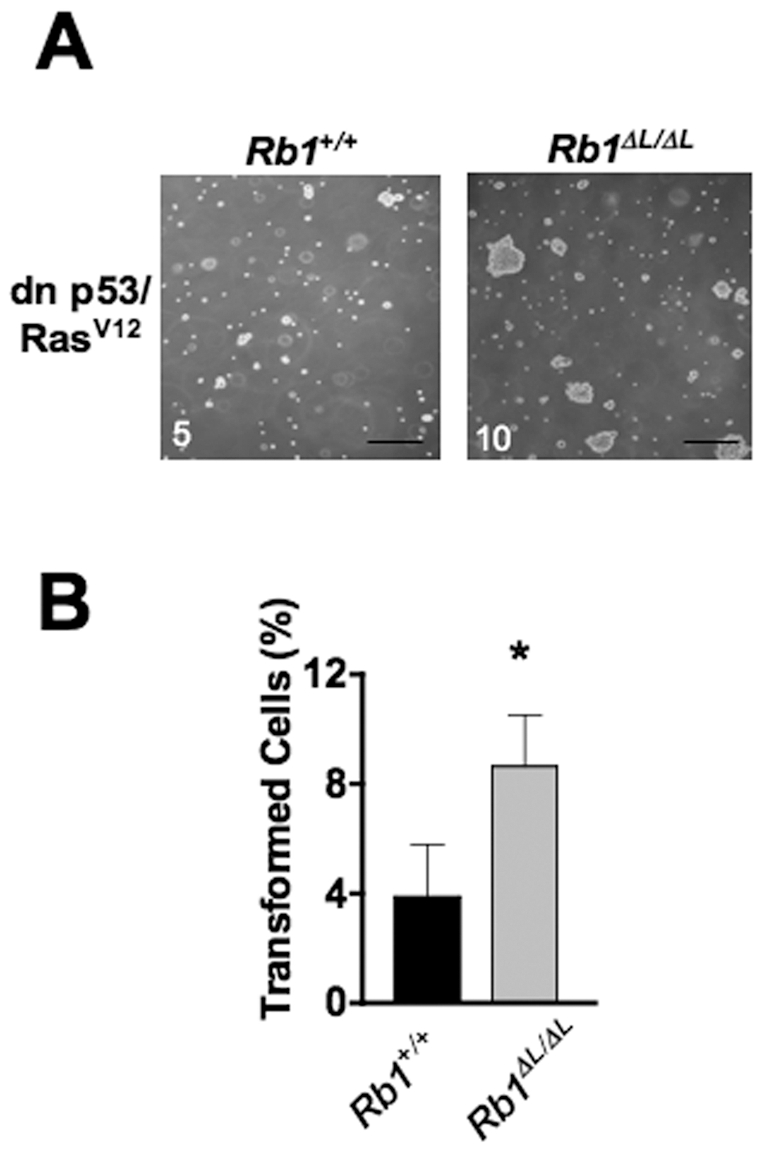
The *Rb1^ΔL^* mutation confers sensitivity to oncogenic transformation. A) MEF cells corresponding to the indicated genotypes were transduced with retroviruses expressing dominant negative (dn) p53 and Ras^V12^ and plated in soft agar to allow colonies to form. Photomicrographs were taken after a two-week growth period. Number in the bottom right corner of each photomicrographs indicate the number of colonies that contained 5 or more cells. Scale: 200 µm. B) The percentage of wild type and mutant cells that transformed and grew into a colony was calculated from five randomly photographed microscopic images. A cell was counted as transformed if it formed a colony whose size appeared to be at least 5 cells. * indicates a statistically significant difference (Student's t test; *P*<0.005). Error bars indicate one standard deviation from the mean for at least three replicates.

Our lab has previously generated a knock-in mouse model (*Rb1^ΔL^*) to disrupt the LXCXE binding cleft on the retinoblastoma tumor suppressor protein [28]. Loss of LXCXE-dependent interactions, between pRB and enzymes such as histone deacetylases, disrupts negative growth control in the mammary gland during development. This leads to hyperplasia of the ductal epithelium that is ubiquitously detectable in virgin animals between four and 16 weeks of age [26]. The *Rb1^ΔL^* mutation alone does not lead to spontaneous mammary cancer, perhaps because this mutation doesn't increase the cellular proliferation rate, but alters the response to negative growth cues [26], [29]. Since hyperplasia of ductal epithelia is considered a risk factor for human breast cancer [30], [31], we postulated that the *Rb1^ΔL^* mutation would exacerbate cancer pathogenesis when combined with other oncogenic changes that provide a proliferative signal. To explore this possibility, we first performed a soft agar colony assay using *Rb1^+/+^* and *Rb1^ΔL/ΔL^* mouse embryonic fibroblasts (MEFs) expressing a dominant negative form of p53 and an oncogenic allele of Ras (pLXSN dn p53/Ras^V12^) [32]. Expression of these oncogenes resulted in a greater frequency of colonies in *Rb1^ΔL/ΔL^* MEFs compared with controls (Fig. 1a, b). Furthermore, many *Rb1^ΔL/ΔL^* colonies were also larger than control colonies (Fig. 1a), suggesting that cells lacking pRB-LXCXE interactions were able to transform earlier. Since the effect of the *Rb1^ΔL^* mutation is exerted after only a few cell divisions, we interpret this phenotype to be caused by a defect in negative growth control rather than genome instability, which can also result from expression of the *Rb1^ΔL^* allele [28], [29]. This provided proof of principle that pRB-LXCXE-dependent anti-growth effects can protect cells from oncogenic transformation.

**Figure 2:**
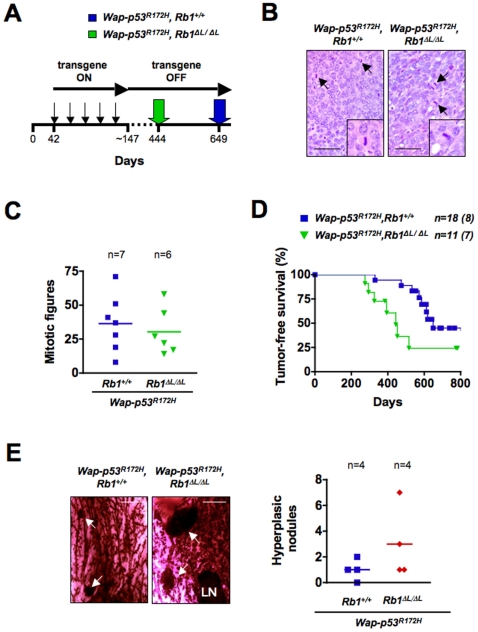
pRB-LXCXE interactions protect against tumor formation caused by the *Wap-p53^R172H^* transgene. A) Experimental outline for the *Wap-p53^R172H^* tumor study. Mice were bred through five rounds of pregnancy (thin arrows) to transiently induce p53^R172H^ expression within the mammary gland. After the fifth pregnancy, males were removed and females were palpated weekly to monitor tumor formation. Median tumor-free survival for each genotype is marked with the colored arrows. B) Representative H&E stained paraffin sections from tumors harvested from *Wap-p53^R172H^*; *Rb1^+/+^* and *Wap-p53^R172H^*; *Rb1^ΔL/ΔL^* mice. Arrows indicate mitotic figures that are shown in the inset. Scale: 50 µm. C) The mitotic index for *Wap-p53^R172H^*; *Rb1^+/+^* and *Wap-p53^R172H^*; *Rb1^ΔL/ΔL^* mice is indicated, along with the average mitotic index for each genotype. Values were derived by quantifying the number of mitotic figures in five random fields of view for each mouse. D) Kaplan-Meier graph of mammary tumorigenesis is shown for *Wap-p53^R172H^*; *Rb1^+/+^* and *Wap-p53^R172H^*; *Rb1^ΔL/ΔL^* females (log rank test; *P* = *0.0238*). Values in brackets indicate the number of mice that developed tumors. E) Carmine Red–stained mammary whole mounts from tumor-free glands in mice that had mammary tumors are shown for both genotypes in the *Wap-p53^R172H^* background. Arrows indicate hyperplastic nodules and LN indicates lymph nodes. Scale: 2 mm. The number of hyperplastic nodules in each whole mount section was also quantified for each genotype along with the average number of hyperplastic nodules.

To validate that pRB-LXCXE interactions can exert a tumor suppressive effect in the mammary gland, we crossed our mice into the *Wap-p53^R172H^* background. *Wap-p53^R172H^* is a dominant negative form of p53 driven by the *whey acidic protein* promoter, which is expressed in the mammary gland during pregnancy and lactation. We bred cohorts of *Wap-p53^R172H^*; *Rb1^+/+^* and *Wap-p53^R172H^*; *Rb1^ΔL/ΔL^* females through five rounds of pregnancy in order to induce transgene expression (Fig. 2a). Since *Rb1^ΔL/ΔL^* females are frequently unable to nurse their pups [26], all live pups were removed two days after birth to ensure consistent timing of transgene expression between genotypes. *Wap-p53^R172H^* expression leads to genomic instability [33], [34], so we reasoned that expression of *p53^R172H^* during pregnancy and lactation would create random mutations. We expected that some of these mutations would drive tumorigenesis later, after the transgene was turned off, and this would allow us to assess how a diminished response to negative growth regulators affects mammary tumorigenesis. Both *Wap-p53^R172H^*; *Rb1^+/+^* and *Wap-p53^R172H^*; *Rb1^ΔL/ΔL^* females developed high grade mammary adenocarcinomas, characterized by high cytological variability. Many cells exhibited large cellular and nuclear size which is comparable to other studies using *Wap-p53^R172H^* mice, indicating that the transgene was active in both genotypes (Fig. 2b) [33], [34]. Furthermore, tumors from both genotypes displayed high but comparable rates of mitosis and similar rates of tumor growth (Fig. 2c and data not shown), demonstrating that the rate of proliferation was similar for *Wap-p53^R172H^*; *Rb1^+/+^* and *Wap-p53^R172H^*; *Rb1^ΔL/ΔL^* tumors.

**Figure 3:**
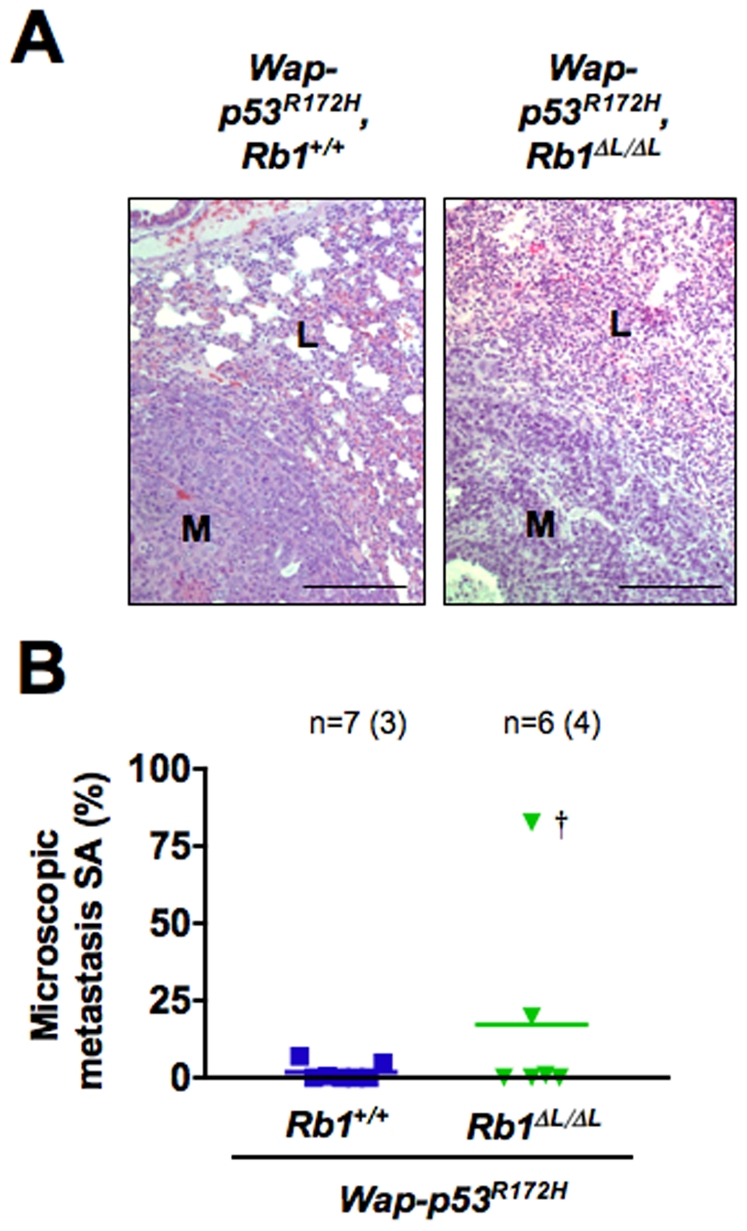
Metastases form in the *Wap-p53^R172H^*; *Rb1* ^+/+^ and *Wap-p53^R172H^*; *Rb1^ΔL/ΔL^* mice. A) Representative H&E stained paraffin sections of lungs harvested from tumor-burdened mice of each genotype. M denotes metastasis, and L denotes neighboring lung tissue. Scale: 100 µm. B) The surface area (SA) occupied by lung metastases relative to the total lung area in tissue sections was quantified for mice from each genotype along with the average SA. Values in brackets indicate the number of mice that developed metastases and † indicates a female that developed metastases to both the lung and spleen.

While mice from both genotypes developed similar types of tumors, we found an increased frequency of tumor formation in *Wap-p53^R172H^*; *Rb1^ΔL/ΔL^* females. 63.6% of *Wap-p53^R172H^*; *Rb1^ΔL/ΔL^* females compared to 44.4% of *Wap-p53^R172H^*; *Rb1^+/+^* females developed tumors over the course of the study. Importantly, loss of pRB-LXCXE interactions in the *Wap-p53^R172H^* background also resulted in earlier tumor onset as detected by mammary palpation (Fig. 2d) (Log rank test, *P* = 0.0238). Like the data from our soft agar colony assay, this suggests that pRB-dependent anti-growth control can act as a barrier to tumor initiation, and when it is lost in *Rb1^ΔL/ΔL^* females *Wap-p53^R172H^* can then drive tumour progression. To explore this concept further, we examined tumor-free mammary glands from our tumor-burdened mice. Some mammary glands had extensive lobuloalveolar development, preventing an assessment of abnormal proliferation by whole mount staining. However, we examined glands where tumors were not palpable among the remaining necropsied animals, and discovered that there was a small increase in the number of hyperplastic lesions in the *Wap-p53^R172H^*; *Rb1^ΔL/ΔL^* mice (Fig. 2e). Together with the increased frequency of tumors and shortened timing of tumor onset in *Wap-p53^R172H^*; *Rb1^ΔL/ΔL^* females, these data indicate that pRB-dependent anti-growth control acts as an initial barrier to tumor formation in the mammary gland. In addition, both *Wap-p53^R172H^*; *Rb1^+/+^* and *Wap-p53^R172H^*; *Rb1^ΔL/ΔL^* tumors were able to metastasize to the lungs, and there were no major differences in the appearance of the metastases, or the number, or size of these metastases (Fig. 3). This is distinct from previous reports demonstrating that loss of pRB-mediated genomic stability leads to more aggressive tumors and increased metastasis [29]. Because the frequency of cancer incidence and age of onset are exacerbated by the *Rb1^ΔL^* mutation, but aspects of progression such as proliferation rate and metastasis are indistinguishable from controls, we conclude that reduced responsiveness to negative growth signals likely functions as a barrier to tumor initiation at a very early stage in cancer pathogenesis.

### Retinoblastoma-dependent negative growth effects on tumorigenesis are context-dependent

The advantage of the *Wap-p53^R172H^* model is that it introduces random mutations and this creates a selection for ones that can cooperate with defects found in *Rb1^ΔL/ΔL^* mice. However, this also prevents us from knowing what the initiating oncogenic mutations were and how they normally engage negative growth responses that activate a pRB-LXCXE-dependent arrest. For this reason we also used a transgenic line that expresses a dominantly acting oncogene so that the origin of oncogenesis would be known. To determine how pRB-dependent responses to negative growth signals affect mammary tumorigenesis in this context, we crossed our mice into the *Tg(MMTVneu)202Mul* (herein referred to as *Neu*) background, where expression of the rat proto-oncogene *Neu* is driven by the *MMTV* promoter. These mice normally develop focal mammary tumors with a high rate of metastasis to the lung [35]. We chose this transgenic line because *Neu* is known to activate the Ras pathway [36] and our data in Figure 1 indicates that pRB-dependent growth arrest opposes it. Furthermore, expression of activated *Neu* in mice with disrupted TGF-β signaling results in reduced tumor latency [37]. Conversely, when crossed to mice that overexpress TGF-β or a constitutively active TGF-β receptor, primary tumor formation is delayed or tumor growth is slowed [37], [38]. This anti-tumor effect is commonly attributed to TGF-β-induced cell cycle arrest, although this aspect of its signaling has not been testable in isolation before now. For these reasons *Neu* mice offer a relatively well characterized system in which to determine how important negative growth signalling is in opposing mammary cancer formation.

**Figure 4:**
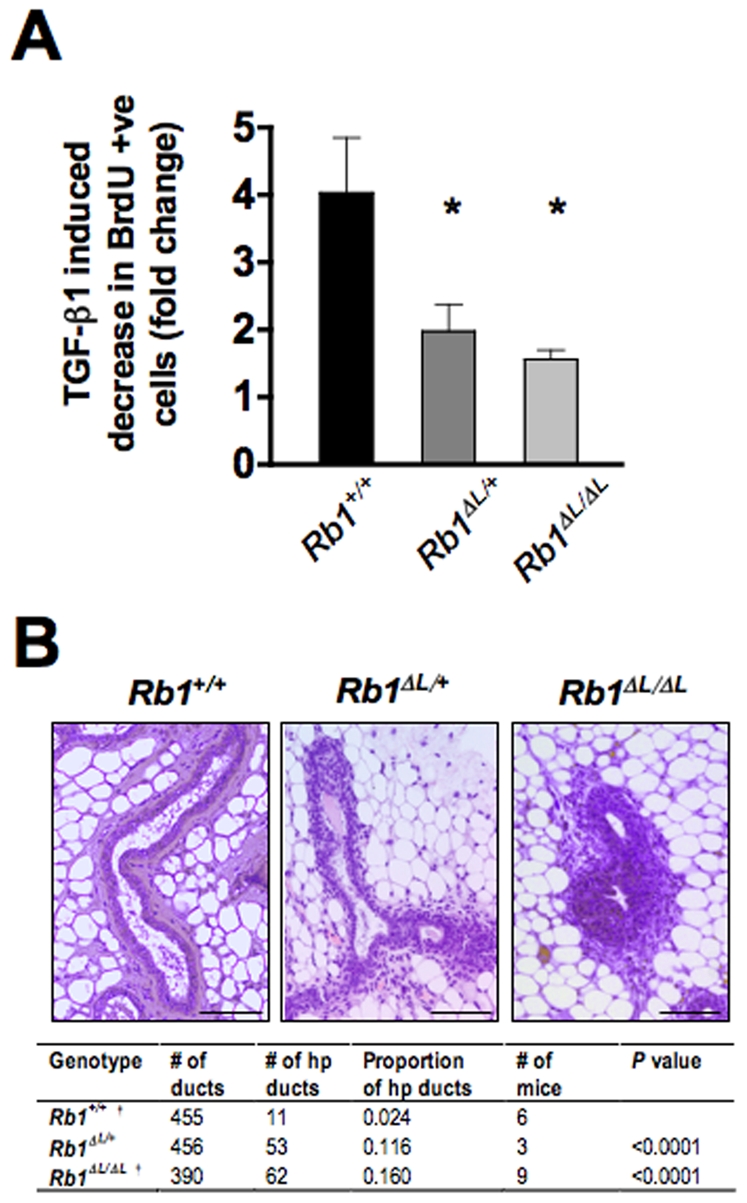
Defective TGF-β proliferative control in *Rb1^ΔL/ΔL^* and *Rb1^ΔL/^* ^+^ mice. A) *Rb1^+/+^*, *Rb1^ΔL/+^*, and *Rb1^ΔL/ΔL^* mammary epithelial cells were treated with TGF-β1 and pulse labeled with BrdU 24 hrs later. The percentage of cells incorporating BrdU was measured by immunofluorescence microscopy. The fold decrease in proliferation between treated and untreated parallel cultures was determined and the average of three independent experiments is shown. * indicates a statistically significant difference (Student's t test; *P*<0.05). Error bars indicate one standard deviation from the mean. B) H&E staining of paraffin sections of *Rb1^+/+^*, *Rb1^ΔL/+^*, and *Rb1^ΔL/ΔL^* mammary tissue from 8 week old mice. Each image displays a representative cross section of ducts. The table below displays the proportion of hyperplastic (hp) ducts found in *Rb1^+/+^*, *Rb1^ΔL/+^*, and *Rb1^ΔL/ΔL^* mammary glands. Proportions were compared between genotypes using a chi-square test. Scale: 100 µm. † denotes previously published data that has been provided for comparison purposes.

Since TGF-β is a key negative regulator of proliferation in the mammary epithelial compartment and induces a G1 arrest in a pRB-LXCXE-dependent manner [26], we next wanted to characterize the *Neu*; *Rb1^ΔL/ΔL^* genotype to ensure that this experimental system would allow us to address the role of negative growth responses during tumorigenesis as we expected. To this end we tested *Rb1^+/+^*, *Rb1^ΔL/ΔL^*, and *Rb1^ΔL/+^* mammary epithelial cells (MECs) for their ability to respond to TGF-β–induced G1 arrest (Fig. 4a). This confirmed our previous results that TGF-β's cytostatic response is vastly diminished in *Rb1^ΔL/ΔL^* cells [26]. Surprisingly, *Rb1^ΔL/+^* MECs have a similar defect in TGF-β growth control indicating that mutation of only one copy of *Rb1* is sufficient to abrogate its arrest mechanism. Consistent with this observation, examination of mammary epithelia in *Rb1^ΔL/+^* virgin female mice revealed they have a similar degree of hyperplasia as we have previously reported for *Rb1^ΔL/ΔL^* mice (Fig. 4b) [26]. The proto-oncogenic *Neu* in these young mice needs to be activated by a deletion in its extracellular domain, so the growth signals that will drive cancer formation are not evident at this early stage [39]. Because of the defective response to negative growth signals caused by the *Rb1^ΔL^* mutation, these mice create an ideal opportunity to assess how an oncogenic growth signal is opposed. Importantly, this can be determined by comparing *Neu* transgenic mice with wild type *Rb1* to either *Rb1^ΔL/+^* or *Rb1^ΔL/ΔL^* animals.

**Figure 5:**
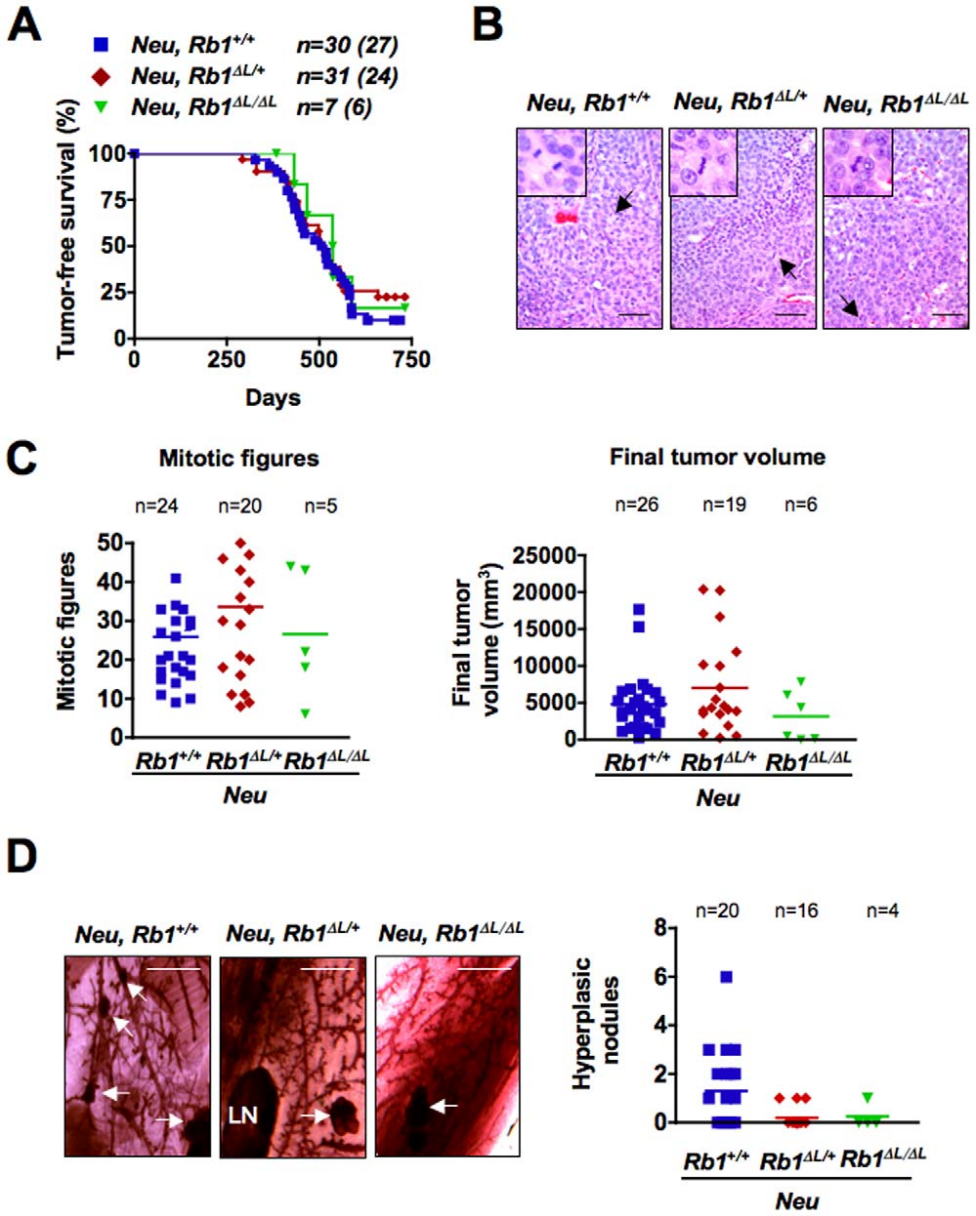
Loss of pRB-LXCXE interactions does not affect *Neu*-driven tumorigenesis. A) Onset of mammary tumorigenesis is shown for the indicated genotypes (log rank test; *P = 0.6788*). Values in brackets indicate the number of mice that developed tumors. B) Representative H&E stained paraffin sections from tumors harvested from *Neu*; *Rb1^+/+^*, *Neu*; *Rb1^ΔL/+^*, and *Neu*; *Rb1^ΔL/ΔL^* mice 60 days after initial tumor palpation. Arrows indicate mitotic figures that are shown in the inset. Scale: 50 µm. C) The mitotic index and final tumor volumes for *Neu*; *Rb1^+/+^*, *Neu*; *Rb1^ΔL/+^*, and *Neu*; *Rb1^ΔL/ΔL^* mice are indicated, along with the average values for each genotype. Mitotic indices were derived by quantifying the number of mitotic figures in five random fields of view for each mouse. Final tumor volume was calculated using the formula V = 0.52×W^2^×L. D) Carmine Red–stained mammary whole mounts from tumor-free glands in mice that had mammary tumors are shown for the given genotypes. Arrows indicate hyperplastic nodules and LN indicates lymph nodes. Scale: 2 mm. The number of hyperplastic nodules in each whole mount section was also quantified for each genotype along with the average number of hyperplastic nodules.

To assess the importance of pRB-dependent negative growth control in suppression of primary tumor formation and growth, we followed cohorts of *Neu*; *Rb1^+/+^*, *Neu*; *Rb1^ΔL/+^* and *Neu*; *Rb1^ΔL/ΔL^* females throughout their natural lives and palpated them weekly to determine the onset of mammary tumor formation. Unfortunately, the long latency before tumor formation resulted in excessive grooming in many of our mice and the need to euthanize them before palpable tumors formed. This was particularly true of the *Neu*; *Rb1^ΔL/ΔL^* mice. However, the *Neu*; *Rb1^ΔL/+^* animals have a similar response to TGF-β, indicating that they offer the same insight into how negative growth signals impact tumorigenesis in this transgenic model. In contrast to the *Wap-p53^R172H^*; *Rb1^ΔL/ΔL^* mice, there was no difference in tumor latency between the remaining *Neu*; *Rb1^ΔL/ΔL^* females and females from the other two genotypes (Fig. 5a). The frequency of tumorigenesis in the *Rb1* mutant genotypes was also relatively unchanged from wild type (85.7% for *Rb1^ΔL/ΔL^* and 77.4% for *Rb1^ΔL/+^* vs. 90% for wild type).

This result suggests that negative growth regulatory signals that depend on pRB do not significantly influence cancer pathogenesis in *Neu* transgenic mice. Because this was unexpected, we also investigated other tumor characteristics to determine if the *Rb1* mutant genotypes altered the tumor type of these mice in such a way that the direct comparison in Fig. 5a is misleading. To this end, we classified the tumors histologically and discovered that they all fit the characteristics of solid or acinar carcinomas that have been reported previously for *Neu* mice, indicating that transgene function was not altered in the different genotypes (Fig. 5b) [40]. Our expectation from the *Wap-p53^R172H^* cross is that negative growth responses are most important at the initiation step. However, Muraoka *et al.* found that overexpression of TGF-β did not affect tumor latency of *Neu* mice, but instead reduced tumor proliferation [38]. For this reason we measured the number of mitotic figures in five randomly selected microscopic fields for each tumor as a means to compare proliferation and this revealed no significant differences (Fig. 5c). Furthermore, there were no significant differences in the final tumor volume (Fig. 5c). Lastly we investigated unaffected mammary glands from tumor-burdened animals for evidence of non-malignant nodules by whole mount preparations. Again, there were no statistically significant differences between the three *Rb1* genotypes and if anything there was a trend toward fewer nodules in mice bearing the *Rb1^ΔL^* mutation (Fig. 5d). These findings are in stark contrast to those for the *Wap-p53^R172H^* cross, where there was an earlier tumor onset in *Wap-p53^R172H^*; *Rb1^ΔL/ΔL^* females and slightly more hyperplastic nodules (Fig. 2a, 2d, 2e). Together, these data indicate that loss of pRB-dependent anti-growth control exacerbates *Wap-p53^R172H^*–dependent tumor formation, but leaves *Neu*-driven tumorigenesis unaltered. This indicates that during cancer formation, the response to anti-growth signals by pRB is context-dependent.

**Figure 6:**
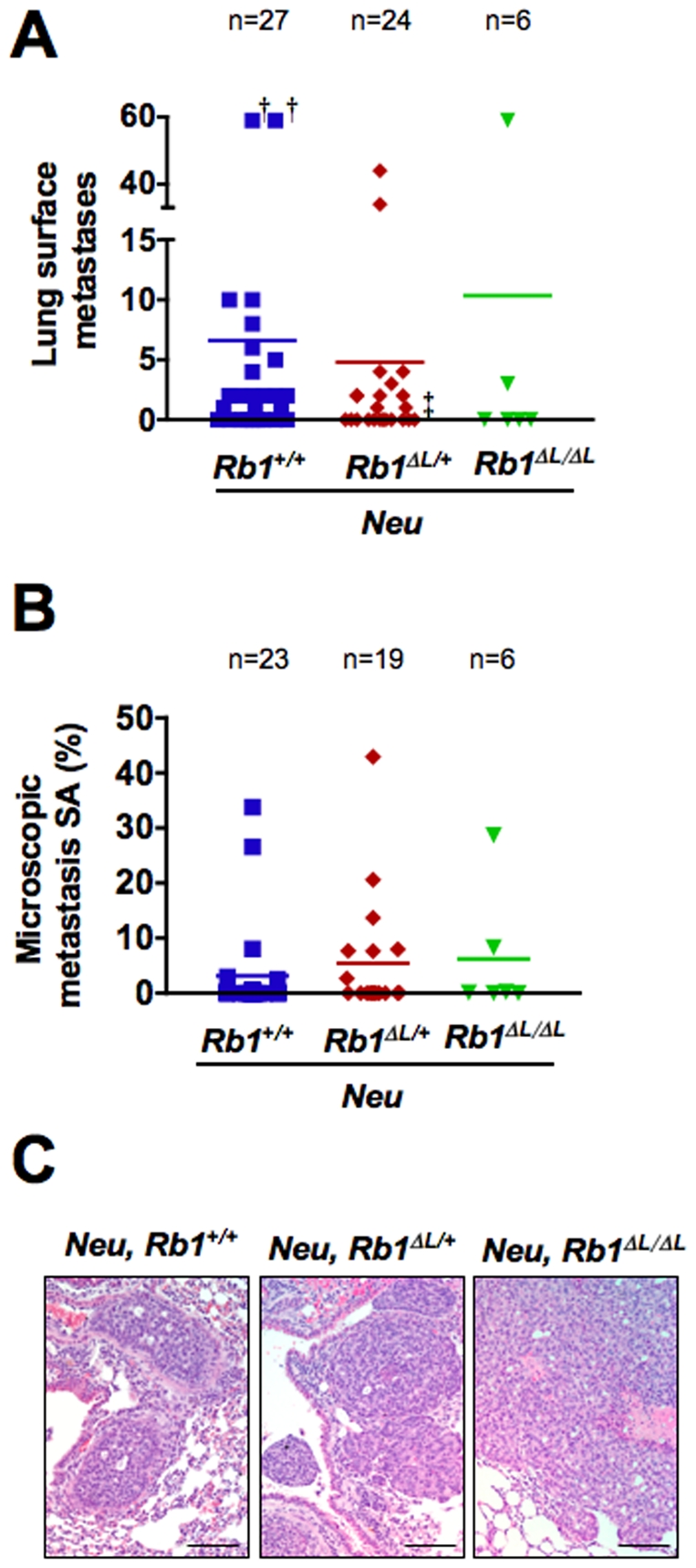
Loss of pRB-LXCXE interactions does not affect metastatic potential in *Neu* mice. A) The number of lung surface metastases (LSM) was quantified for individual mice and plotted along with the average number of LSM per genotype. † indicates lungs with >59 LSM and ‡ indicates a lung where each of the two metastases encompassed an entire lobe. B) The SA occupied by lung metastases relative to the total lung area in tissue sections was quantified for mice from each genotype along with the average SA for each genotype. C) Representative H&E stained paraffin sections of lungs from tumor-burdened mice are shown for each genotype. Scale: 100 µm.

In an effort to better relate the combination of the *Wap-p53^R172H^* and *Neu* transgenes with our *Rb1* mutant, we also investigated metastasis in *Neu*; *Rb1^+/+^*, *Neu*; *Rb1^ΔL/+^*, and *Neu*; *Rb1^ΔL/ΔL^* female mice. This revealed that the number of lung surface metastases that formed during the 60 day period from initial palpation to euthanasia were similar (Fig. 6a). Furthermore, these metastatic lesions occupied a similar proportion of tissue volume when quantified microscopically in lung sections (Fig. 6b). Lastly, there were no apparent differences in histology between metastases from the respective genotypes (Fig. 6c). From these experiments it is clear that the *Rb1^ΔL^* allele does not enhance the metastatic potential of mammary tumors whether they form in the *Neu* or *Wap-p53^R172H^* backgrounds.

This reveals that pRB can confer responsiveness to negative growth signals that limit p53^R172H^ tumorigenic effects. Surprisingly, this aspect of pRB function does not affect *Neu*-driven oncogenesis. Unexpectedly, these experiments reveal that loss of pRB-dependent negative growth responsiveness does not ubiquitously contribute to cancer formation in the mammary gland.

## Discussion

Using two transgenic mouse models of breast cancer, we have examined the importance of pRB-LXCXE interactions during cancer formation and progression. Our work has revealed that LXCXE-dependent anti-growth control can act as a barrier to oncogenic transformation in the mammary gland. Surprisingly, the *Rb1^ΔL^* mutation exacerbated *Wap-p53^R172H^*-induced tumor formation, while having no effect in the *Neu* transgenic background.

The retinoblastoma protein has several important roles in the cell including induction of a cell cycle arrest in response to various stresses, as well as maintaining genome stability [26], [27], [29]. While it is possible that the enhanced tumor phenotype observed here is the result of increased genomic instability, we do not consider this a likely possibility for several reasons. Recent work demonstrated that loss of pRB-mediated genome integrity manifests as more aggressive tumors characterized by altered histopathology compared to controls [29]. Furthermore, they also exhibited increased metastasis [29]. In contrast, we saw no change in tumor size, proliferation, or metastasis in *Wap-p53^R172H^*; *Rb1^ΔL/ΔL^* mice. The increase in tumor penetrance and earlier onset in the *Wap-p53^R172H^*; *Rb1^ΔL/ΔL^* background are more consistent with an inability to induce a growth arrest. We posit that pRB acts as a braking mechanism, protecting the cell from aberrant growth in response to stresses in this transgenic model. Mutations to the LXCXE binding cleft disrupt this braking system, leaving cells vulnerable to oncogenic stimuli, such as those generated in the *Wap-p53^R172H^* model.

The precise pRB LXCXE-dependent anti-growth mechanism that protects against *Wap-p53^R172H^* driven tumors is unclear since mutant p53 acts as a generator of mutations that drive tumorigenesis almost a year after the transgene has been silenced. However, the exact source of anti-growth signals that fail to be received in *Wap-p53^R172H^*; *Rb1^ΔL/ΔL^* mice is not the key question; rather, the *Rb1^ΔL^* mutation allows us to determine the circumstances when the response to negative growth signals are most important. We interpret our data to indicate that pRB responds to these signals to oppose oncogenic transformation in mammary epithelial cells at an early stage in tumorigenesis, before tumors become palpable. Several lines of evidence support this conclusion. First, prior analysis of *Rb1^ΔL/ΔL^* fibroblasts demonstrates that cell size and length of G1 and S phases are similar to wild type cells, indicating that this *Rb1* mutant is phenotypically distinct from the increased rate of proliferation found in *Rb1^−/−^* cells [28]. Instead, *Rb1^ΔL/ΔL^* cells have a defect in inducing cell cycle arrest in response to anti-growth signals (Fig. 4a) [26], [27]. Second, in both the *Wap-p53^R172H^* and *Neu* crosses, the rates of proliferation were comparable between control tumors and those lacking pRB-LXCXE interactions (Fig. 2c and Fig. 5c). In contrast, the median age of tumor onset was significantly lower for *Wap-p53^R172H^*; *Rb1^ΔL/ΔL^* females, compared to control mice (Fig. 2a, 2d). Similarly, only *Wap-p53^R172H^*; *Rb1^ΔL/ΔL^* tumor-free mammary glands displayed an increase in hyperplastic nodule formation. This suggests that the *Rb1^ΔL/ΔL^* mutation impacts tumorigenesis at an early stage, rather than increasing proliferation in existing tumors. Taken together, we interpret these findings to indicate that pRB responds to anti-growth signals to act as a barrier to initial tumor formation by inducing cell cycle arrest; loss of this anti-growth pathway leaves mammary epithelial cells vulnerable to oncogenic transformation caused by p53^R172H^.

Unresponsiveness to negative growth signals is described as a hallmark of cancer, implying a ubiquitous need for it to be eliminated during tumorigenesis [1]. There is little evidence of how these signals are generated and when they act to block aberrant proliferation. The evidence of their importance is largely implied by the frequency of their loss in cell cultures generated from tumors. Our genetic approach is a first step to gaining better insight into this mechanism. Our data from studying *Neu*-induced tumorigenesis implies that negative growth signals may be lost with no apparent effect on cancer incidence. However, comparing the two tumor models used here may be instructive in narrowing down the possibilities. Both *Wap-p53^R172H^* and *Neu* (line 202) require accompanying or sporadic mutations in the transgene to initiate cancer [34], [39]. The *Neu* transgene requires the deletion of a small portion of the ectodomain to be become activate and drive cancer formation. This mechanism has been shown to be consistent between individual *Neu* tumors as similar deletions are generated each time. The resulting activated *Neu* receptors have relatively weak transforming activity in comparison with the original V664E mutant allele [39]. For this reason the relative strength of the oncogenic signal may be an important factor. This is consistent with mammary tumors in *Wap-p53^R172H^*; *Rb1^ΔL/ΔL^* mice having incomplete penetrance; some animals may not acquire sufficiently strong mutations. In addition, mitogenic signals are known to elicit a negative growth response from TGF-β in mammary epithelium [41]. For these reasons the strength of oncogenic growth signal is likely an important cue in determining if a negative growth arrest mechanism will need to be overcome during tumor formation. Future experiments will need to be designed to address this possibility.

Understanding the context in which the response to negative growth signals is a key tumor suppressor mechanism is a challenging new question. However, determining what factors influence context *in vivo* will greatly influence our understanding of negative growth control in cancer, and will undoubtedly impact the classification and treatment of this disease in the future.

## Materials and Methods

### Mouse strains

The *Rb1^ΔL^* mouse strain has been described previously [28]. Analyses of *Rb1^ΔL/ΔL^* mice were performed on a mixed 129/B6 background. *Wap-p53^R172H^* mice were obtained from the Mouse Models of Human Cancer Consortium on an FVB background. These mice express the p53^R172H^ mutation driven by the *whey acidic protein* promoter [33]. These mice were bred to the *Rb1^ΔL^* mutation, creating a mixed 129/B6/FVB genetic background. *Wap-p53^R172H^*; *Rb1^+/+^* and *Wap- p53^R172H^*; *Rb1^ΔL/ΔL^* females were bred through five or six rounds of pregnancy to induce expression of p53^R172H^. Live pups were removed at P2 to allow equivalent timing of transgene expression between genotypes. *Tg(MMTVneu)202Mul* (herein called *Neu*) mice express the wild type form of the rat *Neu* oncogene driven by the *murine mammary tumor virus* promoter (*MMTV*) [35]. These mice were obtained from Jackson Labs on an FVB background and were bred to the *Rb1^ΔL^* mutation, creating a mixed 129/B6/FVB genetic background. Genotyping methods and PCR primers were provided by the suppliers, or are as outlined by Isaac, *et al.* [28]. All animals were housed and handled as approved by the UWO animal use subcommittee (protocol 2007-058) and CCAC guidelines.

### Histology and mammary whole mounts

Full necropsies were performed on tumor-bearing animals after 60 days or at the time of euthanasia. Mammary tumors, lung tissues, and any other tissues that appeared abnormal were fixed in formalin and sectioned as previously described [26]. The mitotic indices were manually counted in 5 high-power fields of view (400×) for mammary tumors from each genotype. Lung metastases were identified by gross morphological analysis (surface metastases) and microscopic analysis (micrometastases). Percent metastatic surface area (SA) was calculated by measuring the total two dimensional area occupied by lung metastases in five hematoxylin and eosin (H&E) stained lung sections, divided by the total area of the lung in these sections using Volocity 4 software (Perkin Elmer, Waltham, MA). Analysis of hyperplasia in H&E stained sections of *Rb1^ΔL/+^* mammary glands, as well as *Neu* expressing mammary glands, was as described before [26]. For whole-mount analysis, unaffected mammary glands from tumor-burdened mice were removed, mounted on glass slides, and stained with Carmine Red using standard techniques.

### Primary cell culture assays

Mammary epithelial cells were harvested and cultured as previously described [26], [42]. Cell culture experiments were carried out on passage 1 or 2 MECs. TGF-β1 growth inhibition assays were performed as previously described [26].

### Soft Agar Colony Formation Assay

*Rb1^+/+^* and *Rb1^ΔL/ΔL^* MEFs were retrovirally transfected with the pLXSN dominant negative (dn) p53/Ras^V12^ virus as previously described [32], [43]. Infected cells were then grown in soft agar according to standard protocols [44]. Cells were allowed to grow for 2 weeks, at which time colonies were photographed and counted. The cut-off for scoring a colony as transformed was that its size needed to correspond with the volume of at least 5 cells (as judged by neighboring single cells). In this way we were confident that these colonies represented multiple cell divisions.

## References

[1] Hanahan D, Weinberg RA (2000). The hallmarks of cancer.. Cell.

[2] Hennighausen L, Robinson GW (2001). Signaling pathways in mammary gland development.. Dev Cell.

[3] Massague J (2004). G1 cell-cycle control and cancer.. Nature.

[4] Sherr CJ (1996). Cancer cell cycles.. Science.

[5] Sherr CJ, Roberts JM (1995). Inhibitors of mammalian G1 cyclin-dependent kinases.. Genes Dev.

[6] Brugarolas J, Chandrasekaran C, Gordon JI, Beach D, Jacks T (1995). Radiation-induced cell cycle arrest compromised by p21 deficiency.. Nature.

[7] Brugarolas J, Moberg K, Boyd S, Taya Y, Jacks T (1999). Inhibition of cyclin-dependent kinase 2 by p21 is necessary for retinoblastoma protein-mediated G1 arrest after gamma-irradiation.. Proc Natl Acad Sci USA.

[8] Florenes VA, Bhattacharya N, Bani MR, Ben-David Y, Kerbel RS (1996). TGF-beta mediated G1 arrest in a human melanoma cell line lacking p15INK4B: evidence for cooperation between p21Cip1/WAF1 and p27Kip1.. Oncogene.

[9] Iavarone A, Massague J (1997). Repression of the CDK activator Cdc25A and cell-cycle arrest by cytokine TGF-beta in cells lacking the CDK inhibitor p15.. Nature.

[10] Missero C, di Cunto F, Kiyokawa H, Koff A, Dotto G (1996). The abscence of p21 Cip1/WAF1 alters keratinocyte growth and differentiation and promotes ras tumor progression.. Genes Dev.

[11] Nakayama K, Ishida N, Shirane M, Inomata A, Inoue T (1996). Mice lacking p27(Kip1) display increased body size, multiple organ hyperplasia, retinal dysplasia, and pituitary tumors.. Cell.

[12] Sharpless NE, Bardeesy N, Lee KH, Carrasco D, Castrillon DH (2001). Loss of p16Ink4a with retention of p19Arf predisposes mice to tumorigenesis.. Nature.

[13] Krimpenfort P, Quon KC, Mooi WJ, Loonstra A, Berns A (2001). Loss of p16Ink4a confers susceptibility to metastatic melanoma in mice.. Nature.

[14] Fero ML, Rivkin M, Tasch M, Porter P, Carow CE (1996). A syndrome of multiorgan hyperplasia with features of gigantism, tumorigenesis, and female sterility in p27(Kip1)-deficient mice.. Cell.

[15] Kiyokawa H, Kineman RD, Manova-Todorova KO, Soares VC, Hoffman ES (1996). Enhanced growth of mice lacking the cyclin-dependent kinase inhibitor function of p27(Kip1).. Cell.

[16] Deng C, Zhang P, Harper JW, Elledge SJ, Leder P (1995). Mice lacking p21CIP1/WAF1 undergo normal development, but are defective in G1 checkpoint control.. Cell.

[17] Krimpenfort P, Ijpenberg A, Song JY, van der Valk M, Nawijn M (2007). p15Ink4b is a critical tumour suppressor in the absence of p16Ink4a.. Nature.

[18] Latres E, Malumbres M, Sotillo R, Martin J, Ortega S (2000). Limited overlapping roles of P15(INK4b) and P18(INK4c) cell cycle inhibitors in proliferation and tumorigenesis.. Embo J.

[19] Robinson GW, Wagner KU, Hennighausen L (2001). Functional mammary gland development and oncogene-induced tumor formation are not affected by the absence of the retinoblastoma gene.. Oncogene.

[20] Fernandez PL, Jares P, Rey MJ, Campo E, Cardesa A (1998). Cell cycle regulators and their abnormalities in breast cancer.. Mol Pathol.

[21] Bosco EE, Knudsen ES (2007). RB in breast cancer: at the crossroads of tumorigenesis and treatment.. Cell Cycle.

[22] Pietilainen T, Lipponen P, Aaltomaa S, Eskelinen M, Kosma VM (1995). Expression of retinoblastoma gene protein (Rb) in breast cancer as related to established prognostic factors and survival.. Eur J Cancer.

[23] Borg A, Zhang QX, Alm P, Olsson H, Sellberg GE (1992). The retinoblastoma gene in breast cancer: allele loss is not correlated with loss of gene protein expression.. Cancer Res.

[24] Nielsen NH, Emdin SO, Cajander J, Landberg G (1997). Deregulation of cyclin E and D1 in breast cancer is associated with inactivation of the retinoblastoma protein.. Oncogene.

[25] Weinberg RA (1995). The retinoblastoma protein and cell cycle control.. Cell.

[26] Francis SM, Bergsied J, Isaac CE, Coschi CH, Martens AL (2009). A functional connection between pRB and transforming growth factor beta in growth inhibition and mammary gland development.. Mol Cell Biol.

[27] Talluri S, Isaac CE, Ahmad M, Henley SA, Francis SM (2010). A G1 checkpoint mediated by the retinoblastoma protein that is dispensable in terminal differentiation but essential for senescence.. Mol Cell Biol.

[28] Isaac CE, Francis SM, Martens AL, Julian LM, Seifried LA (2006). The retinoblastoma protein regulates pericentric heterochromatin.. Mol Cell Biol.

[29] Coschi CH, Martens AL, Ritchie K, Francis SM, Chakrabarti S (2010). Mitotic chromosome condensation mediated by the retinoblastoma protein is tumor-suppressive.. Genes Dev.

[30] Page DL, Dupont WD, Rogers LW, Rados MS (1985). Atypical hyperplastic lesions of the female breast. A long-term follow-up study.. Cancer.

[31] Dupont WD, Page DL (1985). Risk factors for breast cancer in women with proliferative breast disease.. N Engl J Med.

[32] Ossovskaya VS, Mazo IA, Chernov MV, Chernova OB, Strezoska Z (1996). Use of genetic suppressor elements to dissect distinct biological effects of separate p53 domains.. Proc Natl Acad Sci USA.

[33] Li B, Murphy KL, Laucirica R, Kittrell F, Medina D (1998). A transgenic mouse model for mammary carcinogenesis.. Oncogene.

[34] Li B, Rosen JM, McMenamin-Balano J, Muller WJ, Perkins AS (1997). neu/ERBB2 cooperates with p53-172H during mammary tumorigenesis in transgenic mice.. Mol Cell Biol.

[35] Guy CT, Webster MA, Schaller M, Parsons TJ, Cardiff RD (1992). Expression of the neu protooncogene in the mammary epithelium of transgenic mice induces metastatic disease.. Proc Natl Acad Sci USA.

[36] Dankort DL, Wang Z, Blackmore V, Moran MF, Muller WJ (1997). Distinct tyrosine autophosphorylation sites negatively and positively modulate neu-mediated transformation.. Mol Cell Biol.

[37] Siegel PM, Shu W, Cardiff RD, Muller WJ, Massague J (2003). Transforming growth factor beta signaling impairs Neu-induced mammary tumorigenesis while promoting pulmonary metastasis.. Proc Natl Acad Sci USA.

[38] Muraoka RS, Koh Y, Roebuck LR, Sanders ME, Brantley-Sieders D (2003). Increased malignancy of Neu-induced mammary tumors overexpressing active transforming growth factor beta1.. Mol Cell Biol.

[39] Siegel PM, Dankort DL, Hardy WR, Muller WJ (1994). Novel activating mutations in the neu proto-oncogene involved in induction of mammary tumors.. Mol Cell Biol.

[40] Cardiff RD, Anver MR, Gusterson BA, Hennighausen L, Jensen RA (2000). The mammary pathology of genetically engineered mice: the consensus report and recommendations from the Annapolis meeting.. Oncogene.

[41] Ewan KB, Oketch-Rabah HA, Ravani SA, Shyamala G, Moses HL (2005). Proliferation of estrogen receptor-alpha-positive mammary epithelial cells is restrained by transforming growth factor-beta1 in adult mice.. Am J Pathol.

[42] Hojilla CV, Kim I, Kassiri Z, Fata JE, Fang H (2007). Metalloproteinase axes increase beta-catenin signaling in primary mouse mammary epithelial cells lacking TIMP3.. J Cell Sci.

[43] Pear WS, Nolan GP, Scott ML, Baltimore D (1993). Production of high-titre helper-free retroviuses by transient transfection.. Proc Natl Acad Sci USA.

[44] Dannenberg J-H, van Rossum A, Schuijff L, te Riele H (2000). Ablation of the Retinoblastoma gene family deregulates G1 control causing immortalization and increased cell turnover under growth-restricting conditions.. Genes Dev.

